# Counter mucosal incisions: a novel tension-relief technique in anti-reflux mucosal plasty for enhanced closure stability

**DOI:** 10.1055/a-2526-2198

**Published:** 2025-02-11

**Authors:** Mayo Tanabe, Haruhiro Inoue, Kazuki Yamamoto, Kei Ushikubo, Yohei Nishikawa, Boldbaatar Gantuya, Ippei Tanaka

**Affiliations:** 1 378609 Digestive Diseases Center, Showa University Koto Toyosu Hospital, Koto, Japan; 2 26387 Gastroenterology, Sendai Kosei Hospital, Sendai, Japan

We demonstrate the use of counter mucosal incisions as an innovative technique to relieve tension during anti-reflux mucosal plasty with valve (ARM-P/V) ( Video 1 ) 1 . This approach was developed to mitigate the risk of dehiscence at the cardia closure site. ARM-P/V is a minimally invasive procedure for gastroesophageal reflux disease (GERD). This procedure reconstructs the mucosal flap valve to enhance anti-reflux function. The success of ARM-P/V depends on the secure closure of an artificial mucosal defect. However, elevated tension at the closure site may compromise stability, increasing the risk of dehiscence and negatively impacting procedural outcomes.

Counter mucosal incisions were applied during anti-reflux mucosal plasty with valve (ARM-P/V) to relieve tension, preventing dehiscence and improving closure stability in a 38-year-old patient with refractory gastroesophageal reflux disease (GERD).Video 1

In this case, a 38-year-old patient with proton pump inhibitor-refractory GERD underwent ARM-P/V with the application of bilateral counter mucosal incisions ( Fig. 1 **a** ). After creating a mucosal valve using the endoscopic submucosal dissection technique ( Fig. 1 **b** ) and securing the valve with clips ( Fig. 1 **c** ), two linear counter incisions, approximately 3 cm in length, were placed 2 cm lateral to the primary defect ( Fig. 1 **d** ). These incisions effectively relieved tension surrounding the closure site. A submucosal saline injection was administered before making the incisions to elevate the mucosa and protect the underlying muscle layer. Closure of the defect was performed using the dead space-eliminating technique 2 , facilitated by the MANTIS clip (Boston Scientific, Marlborough, Massachusetts, USA), achieving secure mucosal apposition with low tension ( Fig. 1 **e** ). Follow-up endoscopic evaluations on postoperative days 1 and 4 ( Fig. 1 **f** ) confirmed stable closure with no evidence of dehiscence. The procedure was completed without complications, and the incorporation of the counter mucosal incision technique did not prolong the procedural time. This procedure is simple and associated with a low risk of bleeding, making it both safe and efficient.

**Fig. 1:**
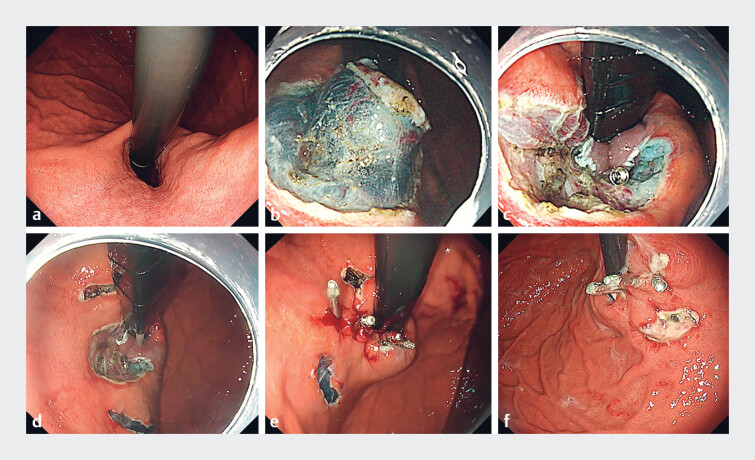
**a** Endoscopic image prior to anti-reflux mucosal plasty with valve showing an open hiatus. **b** Creation of the mucosal valve using the endoscopic submucosal dissection technique. **c** Fixation of the valve with clips. **d** Bilateral counter mucosal incisions to relieve tension. **e** Closure of defect using MANTIS clip. **f** Endoscopic image on postoperative day 4 showing stable closure of the defect.

This novel approach of ARM-P/V draws inspiration from plastic surgery techniques, particularly fasciotomy in compartment syndrome. By applying advanced tissue management principles to high-tension endoscopic closures, counter mucosal incisions represent a promising advancement for achieving stable closures in GERD treatment.

Endoscopy_UCTN_Code_TTT_1AO_2AJ

## Notes

Endoscopy E-Videos https://eref.thieme.de/e-videos
*E-Videos* is an open access online section of the journal *Endoscopy* , reporting on interesting cases and new techniques in gastroenterological endoscopy. All papers include a high-quality video and are published with a Creative Commons CC-BY license. Endoscopy E-Videos qualify for HINARI discounts and waivers and eligibility is automatically checked during the submission process. We grant 100% waivers to articles whose corresponding authors are based in Group A countries and 50% waivers to those who are based in Group B countries as classified by Research4Life (see: https://www.research4life.org/access/eligibility/ ). This section has its own submission website at https://mc.manuscriptcentral.com/e-videos .
